# Socialising over fruits and vegetables: the biocultural importance of an open-air market in Bandar Seri Begawan, Brunei Darussalam

**DOI:** 10.1186/s13002-020-0356-6

**Published:** 2020-01-31

**Authors:** F. Merlin Franco, Li Ling Chaw, Nurzahidah Bakar, Siti Noraqilah Haji Abas

**Affiliations:** 1 Institute of Asian Studies, Universiti Brunei Darussalam, Jalan Tungku Link, Gadong, BE1410 Brunei Darussalam; 2 Pengiran Anak Puteri Rashidah Sa’adatul Bolkiah Institute of Health Sciences, Universiti Brunei Darussalam, Jalan Tungku Link, Gadong, BE1410 Brunei Darussalam; 3 Universiti Brunei Darussalam, Jalan Tungku Link, Gadong, BE1410 Brunei Darussalam

**Keywords:** Borneo, Southeast Asia, Biodiversity, Endemic, Ethnicity, Culture, Plant blindness

## Abstract

**Background:**

Earth’s biocultural diversity comprising biological, cultural and linguistic diversities is being eroded quickly. Our ability to recognise and appreciate what is remaining is crucial for its survival. However, not all forms of diversity are appreciated equally and a growing trend in plant blindness indicates that humans ignore plants in the environment. In this context, open-air markets emerge as cultural spaces that bring people closer to each other, as well as with local biodiversity represented by fruits, vegetables and medicinal plants.

**Methods:**

We conducted a cross-sectional survey with 160 people visiting Tamu Kianggeh of Bandar Seri Begawan, Brunei Darussalam. We randomly interviewed every fifth adult visitor (> 18 years) leaving the market on Fridays and Sundays continuously for a month, using a structured questionnaire. The questionnaire had 18 questions related to demographic particulars, reasons for visiting the market, vendor preference, social networking and visits to open-air markets and supermarkets.

**Results and discussion:**

People visit the market for the diversity of vegetables/fruits; local fruits and vegetables; socialising; cheap prices; ability to bargain; freshness of the products; convenience; medicinal plants; snacks; leisure etc. The ethnic diversity represented at the market comprised chiefly of Malay, Kedayan, Iban, Dusun, Tutong, Chinese communities and foreigners. Majority of the respondents chose ‘availability of a wide range of fruits and vegetables’ as the primary reason for the visit, followed by ‘availability of local fruits and vegetables’. Tamu Kianggeh sold larger number of fruits and vegetables (104 taxa, 26 natives, 2 endemics) compared to the nearest supermarket (85 taxa, 14 natives and 1 endemic). A significant number of respondents also reported that they had made friends at the market.

**Conclusion:**

Tamu Kianggeh is a meeting ground for ethnic and biological diversities, a property that makes them important centres of biocultural diversity at the local level. Open-air markets such as Tamu Kianggeh bring people closer to a diverse range of vegetables and fruits. They also bring people closer to each other by serving as platforms for socialising. We propose that strategies developed to counter plant blindness should also consider the potential of open-air markets.

## Background

Biocultural diversity (BCD) is defined as the sum total of the diversity of life in all of its manifestations: biological, cultural and linguistic, which are interrelated (and possibly coevolved) within a complex socioecological adaptive system [1]. This diversity comprising biological diversity and ‘human beliefs, values, worldviews and cosmologies’ is being lost at alarming rates [2, 3]. The survival of earth’s remaining biocultural diversity depends on our ability to recognise and appreciate it. However, not all forms of diversity are recognised and appreciated equally. Unlike animals that are considered charismatic and appreciated, plants and their produces are often overlooked. This phenomenon is termed as ‘plant blindness’, a term coined by Wandersee and Schussler [4]. Bringing people closer to plants and the associated culture is imperative to address plant blindness and conserve biocultural diversity [5]. The concept of BCD draws strength largely from studies that have explored the co-occurrence of biodiversity and linguistic diversity [3], biocultural approaches to conservation [6], importance of traditional knowledge and management regimes in biodiversity conservation [7], ecological and cultural importance of species [8] and the importance of biocultural landscapes such as sacred groves and sacred natural sites [9]. However, studies approaching open-air markets from the biocultural diversity perspective are lacking. In this article, we use a case study from Bandar Seri Begawan to highlight the biocultural value of open-air markets as centres of confluence of biodiversity and ethnic diversity. We propose that this unique ability of markets to bring people closer to each other, as well as to biodiversity, could make them excellent avenues for combating plant blindness.

### Defining open-air markets

Open-air markets are considered one of the earliest forms of trade centres, where exchange occurs between buyers and sellers. Colloquially, they are known by various names such as flea markets, swap meets, rural markets, farmers’ markets, peasant markets, periodic markets, wet markets and trading fairs [10]. Though these names are often used interchangeably, they refer to market places of varying nature with reference to periodicity, purpose, magnitude of trade, location etc. The term ‘markets’ itself is ambiguous as it can refer to exchanges or the ‘social relationships and frameworks’ that facilitate economic transmissions, or geographically specific social institutions, with ‘specific social, legal, and political processes that enable economic transactions’ [11]. Plattner, however, navigated around this confusion by referring to ‘markets’ as institutions of exchanges and distinguishing them from ‘marketplaces’ that are geographical locations where exchanges occur [12]. Bestor while discussing the anthropological aspects of markets recognises the term ‘market’ to have dual sense, both marketplaces, as well as markets, and uses the term market to refer to marketplaces [11]. For the purpose of this paper, we use the terms ‘market’ and ‘open-air markets’ interchangeably to refer to marketplaces.

### Origin and characteristics of open-air markets

Markets are thought to have appeared with the birth of settled agriculture. The earliest known markets are believed to have originated 5000 years ago in the Fertile Crescent [13]. Some of the contemporary markets have a long history of existence, while other prominent ones have disappeared over time. It is now understood that the present day Mayan marketplaces are continuums from pre-capitalistic times [14], and marketplaces played an important role in sustaining the erstwhile Mayan civilization [15]. In ancient India, markets such as the Arikamedu served as an important nodal point for overseas trade [16]. Skinner’s study with the markets of rural China shows that markets and market towns were ‘central places’ [17]. The outward movement of agricultural and craft goods produced in the local area began from these central places, while the flow of imported goods meant for the consumption of peasants ended there. According to Rozelle et al., the traditional markets in contemporary rural China have retained much of their traditional characteristics [18]. In Thailand, markets are known to be dynamic entities that adapt to changing times leveraging on the multiple networks including tourism [19].

### Prior studies on open-air markets

Owing to the confluence of people, culture, biodiversity, languages and even germs, markets attract researchers from various disciplines to date. A notable work from Southeast Asia is that of Alexander [20], who provides a comprehensive understanding of the vendors of the peasant market system in Kebumen, Java. Her work deals with the geographical location and distribution, types of markets, the kinds of traders, flow of information in market and the social relationships that exist in markets. Anthropologists have studied the relationship between markets and marketplaces, the ethnographic values, the social and governance structures, cultural patterns, relationship between cities and their markets, and globalisation points of view [11]. Geographers have studied the spatial organisation and the temporality of markets [21]. Economists have studied people’s preferences for purchasing at the markets [22, 23]. Ethnobiologists have studied the diversity of medicinal plants traded, their cultural values and knowledge on medicinal plants in markets [24–26]; from the biodiversity and nutrition perspective, researchers have studied the sale of wildlife [27], diversity of edible plants and fungi [28, 29] and contribution to dietary diversity [30]. When unregulated, markets are also known to fuel trade in rare and endemic species, which in turn could lead to the decline of wild populations [31, 32]. Hence, researchers have routinely inventoried markets to monitor the trade in rare and endemic taxa [29, 33]. Microbiologists have investigated microbial quality of produces sold in the markets [34], drug resistance in microbes found in the markets etc. [35]. There are even studies that have adopted a psychological approach such as understanding the impact of background music on consumer behaviour in markets [36]. The above list indicates the various disciplinary approaches researches have adopted to study markets. However, it also indicates that the overall biocultural importance of markets has been overlooked.

## Methods

This study deals with Tamu Kianggeh, an open-air market in Bandar Seri Begawan (BSB), the capital city of Brunei Darussalam. BSB is one of the smallest cities in Asia. It has a total of two open-air markets, viz. Tamu Kianggeh and Tamu Gadong. This study only involved Tamu Kianggeh as we were not successful in securing permission to conduct interviews at Tamu Gadong. We conducted a cross-sectional quantitative survey of people visiting Tamu Kianggeh to uncover the reasons behind their patronage for open-air markets despite the availability of supermarkets close by. Following it, we undertook a onetime inventorying of fruits and vegetables sold in the market and the nearest supermarket to generate a quick understanding of the range of fruits and vegetables available to people through these markets. We use the results of these surveys to understand the biocultural importance of the market. The following sections explain in detail the methodology adopted for this study.

### Characteristics of Tamu Kianggeh

**Fig. 1:**
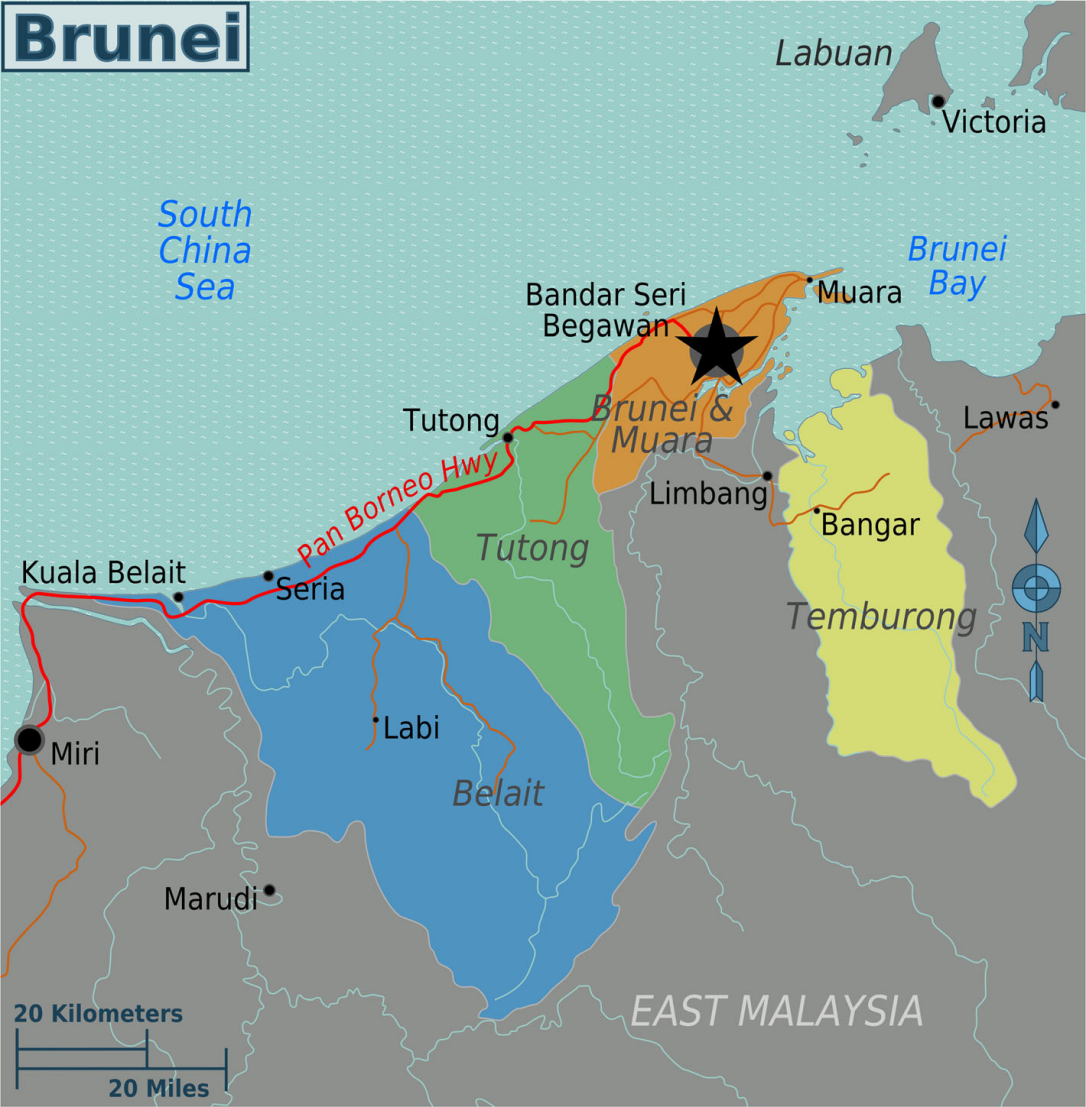
Map of Brunei Darussalam showing Bandar Seri Begawan. File credits: Creative Commons CC BY-SA 3.0 US [37]

**Fig. 2:**
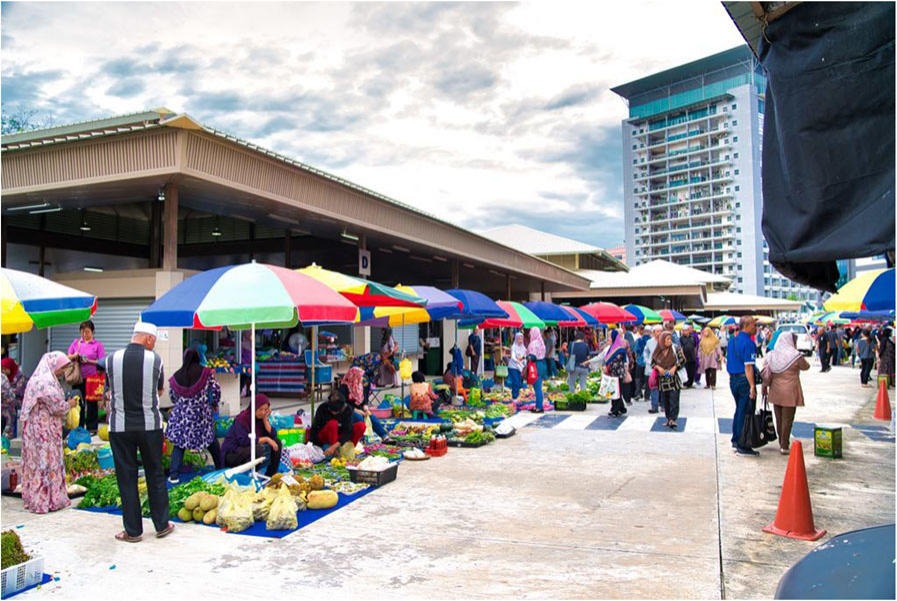
Vendors selling produces outside the tin-roofed area. Credits: F. Merlin Franco

Tamu Kianggeh is located in BSB, the capital of Brunei Darussalam (Fig. 1). It originated as an open-air market and still retains its nature despite a tin roof. The market is also a periodic market that convenes on the forenoons of Fridays and Sundays and has two entry and exit points, one each at the eastern and westerns faces, respectively. It is largely a wet market with the most number of vendors selling vegetables and fruits, followed by fish, snacks, fowls and handicrafts. On a Friday, we counted 168 vendors selling fruits and vegetables, 36 food vendors, 26 fish vendors and six handicrafts sellers (Fig. 2). Besides, there are also kiosks selling plastic toys and utensils, dry food and provisions.

### Sampling and interviews

In this study, the eligible questionnaire respondents are people other than vendors above the age of 18, visiting Tamu Kianggeh with an intention of making a purchase or to inventory the materials available for purchase. All authors are frequent visitors of the market. Information collected through observation and casual conversations with vendors and fellow visitors were used to design the questionnaire. We observed that visitors do not stay back for a prolonged time post-visit, and hence the questionnaire had to be kept short. There were 18 questions in total (Table 2), questions 1 to 7 were on demographic particulars; 8 and 9 were on reasons for visiting the market; 10 on vendor preference; 11, 12, 13 and 14 on social networking; and 15 to 18 on the visit to markets and supermarkets and the groceries available there. Question 5 was on ethnicity of the respondent—if they identify themselves as a member of a community indigenous to the larger Borneo island. The paper-based questionnaire was then pilot tested initially by administering it to friends and relatives, followed by pilot interviews at the Kianggeh market on the 6th and 8th of April 2018. During the course of the pilot interviews, we discovered that there was a possibility for the economic status of visitors to differ depending on which of the two entry/exit points they used. The eastern entrance had ample parking spaces and hence was preferred by visitors who arrived by cars, whereas the western gate was preferred by visitors who used public transport. This is because of the proximity of the public bus station to the western entrance. Due to this, the interviews were held at alternating gates every week. Interviews were conducted on all Fridays and Sundays from the 13th of April 2018 to 6th May 2018. Twenty randomly chosen respondents were interviewed each day, between 7.00 and 12.00 h., both for the pilot testing as well as the interviews. In total, we gathered 160 respondents. The socio-economic profile of the respondents is provided in Table 1.

**Table 1:** Demographic and socio-economic profile of respondents

Number of respondents	160 (males = 83; females = 77)	
Age group	20–39	49
	40–64	97
	65 and above	14
Nature of residence	Locals	110
	Foreigners	50
Whether a member of indigenous community (self-identification)	Indigenous	87
	Non-indigenous	73
Monthly income (Brunei Dollars)	Less than 1000	90
	1000–1999	35
	2000–2999	18
	Above 3000	12
	Not available	5
Education level	None	0
	Primary	13
	Secondary	95
	Vocational/diploma	4
	Undergraduate	21
	Postgraduate and above	6
	Not available	21

A systematic random sampling approach was used, where every fifth adult visitor (irrespective of gender) leaving the market was approached for the study. In situations where the fifth visitor refused to be interviewed or has been interviewed previously, the interviewers moved on to the tenth visitor and so on. Finding respondents was not easy, as learnt from our experience with the pilot interviews. This is because visitors were keen on getting back home immediately post-visit. Lack of time was the most frequently cited reason for declining to be interviewed, followed by hot weather. There were a few migrant labourers who were interested in responding but had to decline due to language barriers. One respondent refused to be interviewed citing ‘lack of teeth’ indicating the role of personal confidence. The respondent data is anonymous; thus, no data can be individually linked to the personal identity of the respondent. Descriptive analyses were carried out and group comparison tests (chi-square and Fisher’s exact) were used to determine any group differences between indigenous and non-indigenous, and leisure and non-leisure market visitors, as well as cross-comparisons between responses and demographic particulars. All analyses were done using R (ver.3.5.1) statistical software. A *p* value of < 0.05 was considered statistically significant. Risk assessment was conducted prior to the commencement of the survey, and ethical clearance was obtained from University Research Ethics Committee, Universiti Brunei Darussalam (UBD/OAVCR/UREC/Dec17-01). Permission to conduct interviews at the market was obtained from the Jabatan Bandaran, BSB. The research conforms to the code of ethics of the International Society of Ethnobiology [38].

### Inventorying of vegetables and fruits

Following the compilation of the findings, a onetime inventorying of the market and the nearest supermarket was conducted to compare the findings of the interviews with the availability of vegetables and fruits. The nearest supermarket that is large enough to be considered a representative of the supermarkets in Brunei is approximately 3 km away from Tamu Kianggeh. Common names of the vegetables and fruits sold in the supermarket were noted down from the labels, while in the market, we enquired the vendors for the local name. The vegetables and fruits were initially identified provisionally using a checklist created by the last two authors for a different project and later confirmed by referring to Hutton’s Tropical vegetables [39] and Det et al.’s Edible wild plants in Sarawak [40], as well as by comparing with specimens at the Brunei National Herbarium (BRUN). Latin names of the plants and their biogeography were updated by referring to online databases and published literature [41–45]. There were also dry medicinal plant materials such as roots and barks sold in the market. However, we had excluded them from the current study as identifying them would require detailed analyses at the anatomical and molecular levels [46].

## Results and discussion

Majority of respondents (*n* = 97, 60.6%) were of the age group 40–64 years, followed by the age group 20–39 years (*n* = 49, 30.6%) (Table 1). There were 14 senior citizens above the age of 65 (8.8%). Most of our respondents were married (*n* = 129, 80.6%). With regard to the residency status, there were 110 locals (*n* = 110, 68.8%) and the rest were foreigners (*n* = 50, 31.2%). Income wise, more than half of the respondents (*n* = 90, 56.2%) reported a monthly income of less than 1000 Brunei dollars (BND), followed by people earning 1000–1999 BND (*n* = 35, 21.9%). The number of visitors progressively gets lower as the income increases. Among the respondents who reported purchasing regularly from supermarkets, 42 (46.6%) of them had a monthly income of more than 2000 BND, followed by those earning less than 1000 BND (*n* = 30, 33.3%) and 1000–1999 BND (*n* = 18, 20.0%). The popular notion is that rich consumers prefer to shop from supermarkets [47]. However, among respondents who earned more than 2000 BND, there was no significant difference between those who purchased regularly from the supermarket and those who did not. This indicates that income is not the primary factor that determines visitors’ preferences for open-air markets or supermarkets. The raw data from the interviews is available as Table 2.

**Table 2:** Raw data from the interviews

Characteristics		Total respondents	%	Visit the Tamu leisurely (*n* = 51)	%	Do not visit the Tamu leisurely (*n* = 109)	%	*p* value^^^	Indigenous (*n* = 87)	%	Non-indigenous (*n* = 73)	%	*p* value^^^
Gender	Male	83	51.9	29	56.9	54	49.5	0.488	47	46	36	49.3	0.664
	Female	77	48.1	22	43.1	55	50.5		40	54	37	50.7	
Age	20–39	49	30.6	20	39.2	29	26.6		22	25.3	27	37	
	40–64	97	60.6	25	49	72	66.1	0.122	54	62.1	43	58.9	0.073
	65 above	14	8.8	6	11.8	8	7.3		11	12.6	3	41.1	
Marital status	Married	129	80.6	39	76.5	90	82.6	0.487	75	86.2	54	74	0.08
	Single	31	19.4	12	23.5	19	17.4		12	13.8	19	26	
Residential status	Local	110	68.8	37	72.5	73	67	0.599	84	96.6	26	35.6	< 0.001*
	Foreigner	50	31.2	14	27.5	36	33		3	3.4	47	64.4	
Period of Stay	Tourist	2	4.0	1	7.2	1	2.8						
	< 1 year	1	2.0	0	0	1	2.8	0.12					
	1–5 years	9	18.0	5	35.7	4	11.1						
	> 5 years	38	76.0	8	57.1	30	83.3						
Frequency of market visit in native country	Regularly	34	68.0	7	50	27	75						
	Sometimes	13	26.0	6	42.9	7	19.4	0.154					
	No	3	6.0	1	7.1	2	5.6						
Frequency of Tamu visit	Regularly	101	63.1	29	56.9	72	66.1		49	56.3	52	71.2	
	Sometimes	56	35.0	20	39.2	36	33	0.224	38	43.7	18	24.7	0.008*
	Only during festival	0	0.0	0	0	0	0		0	0	0	0	
	Others	3	1.9	2	3.9	1	0.9		0	0	3	4.1	
Are you from an indigenous community?	No	73	45.6	22	43.1	51	46.8	0.793					
	Yes	87	54.4	29	56.9	58	53.2						
If yes, which group are you from?	Malay	58	66.7	18	62	40	69						
	Kedayan	17	19.5	7	24.1	10	17.2						
	Dusun	3	3.5	1	3.5	2	3.5	0.211					
	Iban	5	5.7	0	0	5	8.6						
	Melayu Tutong	3	3.5	2	6.9	1	1.7						
	Serawak Kuching	1	1.1	1	3.5	0	0						
Monthly Income	< BND1000	90	56.2	28	54.9	62	56.9		48	55.2	42	57.5	
	BND1000–1999	35	21.9	11	21.6	24	22	0.592	22	25.3	13	17.8	0.492
	BND2000–2999	18	11.3	8	15.7	10	9.2		10	11.5	8	11	
	> BND3000	12	7.5	2	3.9	10	9.2		6	6.9	6	8.2	
	Not available	5	3.1	2	3.9	3	2.7		1	1.1	4	5.5	
Education level	None	0	0.0	0	0	0	0		0	0	0	0	
	Primary	13	8.1	6	11.8	7	6.4		6	6.9	7	9.6	
	Secondary	95	59.4	26	51	69	63.3	0.395	57	65.5	38	52	0.005*
	Vocational/diploma	25	15.6	11	21.5	14	12.8		17	19.5	8	11	
	BA	21	13.1	6	11.8	15	13.8		7	8.1	14	19.2	
	MA and above	6	3.8	2	3.9	4	3.7		0	0	6	8.2	
Main reason for visiting Tamu	Cheap prices	16	10.0	5	9.8	11	10.1		8	9.2	8	10.9	
	Lots of vegetables/fruits	90	56.3	31	60.8	59	54.1		45	51.7	45	61.6	
	Local fruits and vegetables	18	11.3	5	9.8	13	11.9	0.729	13	14.9	5	6.9	0.508
	Meeting familiar people	0	0.0	0	0	0	0		0	0	0	0	
	Can bargain	1	0.6	0	0	1	0.9		1	1.2	0	0	
	Fresh	11	6.9	2	3.9	9	8.3		6	6.9	5	6.9	
	Convenient	4	2.5	0	0	4	3.7		1	1.2	3	4.1	
	Medicinal plants	0	0.0	0	0	0	0		0	0	0	0	
	Snacks	10	6.2	5	9.8	5	4.6		7	8	3	4.1	
	Others	10	6.2	3	5.9	7	6.4		6	6.9	4	5.5	
Other reasons for visiting Tamu^#^	Cheap prices	91	56.9	28	54.9	63	57.8		51	58.6	40	54.8	
	Lots of vegetables/fruits	112	70.0	40	78.4	72	66.1		57	65.5	55	75.3	
	Local fruits and vegetables	79	49.4	24	47.1	55	50.5		35	40.2	44	60.3	
	Meeting familiar people	40	25.0	15	29.4	25	22.9		21	24.3	19	26	
	Can bargain	38	23.8	12	23.5	26	23.9		15	17.2	23	31.5	
	Fresh	78	48.8	25	49	53	48.6		32	36.8	46	63	
	Convenient	73	45.6	23	45.1	50	45.9		26	29.9	47	64.4	
	Medicinal plants	20	12.5	8	15.7	12	11		11	12.6	9	12.3	
	Snacks	75	46.9	25	49	50	45.9		26	41.4	39	53.4	
	Others	7	4.4	3	5.9	4	3.7		5	5.7	2	2.7	
Whom did you purchase from?	Regular vendor	65	40.6	14	27.5	51	46.8		33	37.9	32	43.8	
	Whoever offers cheap prices	27	16.9	11	21.5	16	14.7	0.066	16	18.4	11	15.1	0.718
	Any vendor offering clean and healthy product	68	42.5	26	51	42	38.5		38	43.7	30	41.1	
Do you visit Tamu with friends?	Yes	22	13.8	8	15.7	14	12.9		9	10.3	13	17.8	
	Sometimes	41	25.6	17	33.3	24	22	0.214	20	23	21	28.8	0.195
	No	97	60.6	26	51	71	65.1		58	66.7	39	53.4	
Have you made friends at the Tamu?	Yes	97	60.6	28	54.9	69	63.3	0.401	61	70.1	36	49.3	0.012*
	No	63	39.4	23	45.1	40	36.7		26	29.9	37	50.7	
If yes, with whom did you make friends with?	Vendors	84	86.6	28	100	56	81.2		53	86.9	31	86.1	
	Visitors	32	33.0	10	35.7	22	31.9		17	27.9	15	41.7	
	Others	4	4.1	0	0	4	5.8		3	4.9	1	2.8	
Do you purchase groceries as a favour for your neighbour?	Yes, often	23	14.4	8	15.7	15	13.8		15	17.2	8	11	
	Yes, sometimes	48	30.0	17	33.3	31	28.4	0.72	28	32.2	20	27.4	0.322
	No	89	55.6	26	51	63	57.8		44	50.6	45	61.6	
Would you visit the Tamu even if you don't have a purchase to make?	Yes	51	31.9						29	33.3	22	30.1	0.793
	No	109	68.1						58	66.7	51	69.9	
How often do you purchase from supermarket?	Regularly	64	40.0	20	39.2	44	40.4		38	43.7	26	35.6	
	Yes, only if it is not available in Tamu	28	17.5	6	11.8	22	20.2	0.457	15	17.2	13	17.8	0.573
	Rarely	67	41.9	25	49	42	38.5		34	39.1	33	45.2	
	Never	1	0.6	0	0	1	0.9		0	0	1	1.4	
Should every city have local markets?	Yes	153	95.6	49	96	104	95.4		86	98.8	67	91.8	
	No	4	2.5	1	2	3	2.8	1	1	1.2	3	4.1	0.081
	Do not know	3	1.9	1	2	2	1.8		0	0	3	4.1	
Do you think there are groceries only in local market?	Yes	81	50.6	26	51	55	50.5	1	53	60.9	28	38.4	0.007*
	No	79	49.4	25	49	54	49.5		34	39.1	45	61.6	
If yes, name some of these groceries	Local goods	54	81.1	16	69.6	38	88.4		37	86	17	73.9	
	Traditional goods	13	19.7	6	26.1	7	16.3		8	18.6	5	21.7	
	Traditional medicines	6	9.1	4	17.4	2	4.7		3	7	3	13	
	Fish/seafood	11	16.7	7	30.4	4	9.3		5	11.6	6	26.1	
	Live poultry	3	4.5	2	8.7	1	2.3		2	4.7	1	4.3	
	Beverage	2	3.0	1	4.3	1	2.3		1	2.3	1	4.3	
	Nuts and legumes	1	1.5	0	0	1	2.3		1	2.3	0	0	
Do you think there are groceries only in supermarket?	Yes	86	53.8	23	45.1	63	57.8	0.183	53	60.9	33	45.2	0.068
	No	74	46.2	28	54.9	46	42.2		34	39.1	40	54.8	
If yes, name some of these groceries	Imported goods	33	47.1	9	50	24	46.2		20	48.8	13	44.8	
	Processed goods	33	47.1	7	38.9	26	50		19	46.3	14	48.3	
	Poultry	15	21.4	3	16.7	12	23.1		9	22.2	6	20.7	
	Local products	2	2.9	0	0	2	3.8		2	4.9	0	0	
	Grain products	3	4.3	1	5.6	2	3.8		2	4.9	1	3.4	
	Meat	9	12.9	2	11.1	7	13.5		6	14.6	3	10.3	
	Eggs	1	1.4	1	5.6	0	0		1	2.4	0	0	
	Condiments/seasoning/spices	2	2.9	2	11.1	0	0		2	4.9	0	0	
	Dairy products	2	2.9	0	0	2	3.8		1	2.5	1	3.4	
	Fish	2	2.9	0	0	2	3.8		1	2.5	1	3.4	
	Nuts/legumes/by-product	1	1.4	0	0	1	1.9		1	2.5	0	0	
Gate where interview took place	West	80	50.0	23	45.1	57	52.3	0.497	46	52.9	34	46.6	0.526
	East	80	50.0	28	54.9	52	47.7		41	47.1	39	53.4	

^#^Multiple responses were allowed

^^^Derived from independent chi-square or Fisher's exact test (whichever is appropriate)

*p value is statistically significant (< 0.05)

### Markets and biodiversity

**Fig. 3:**
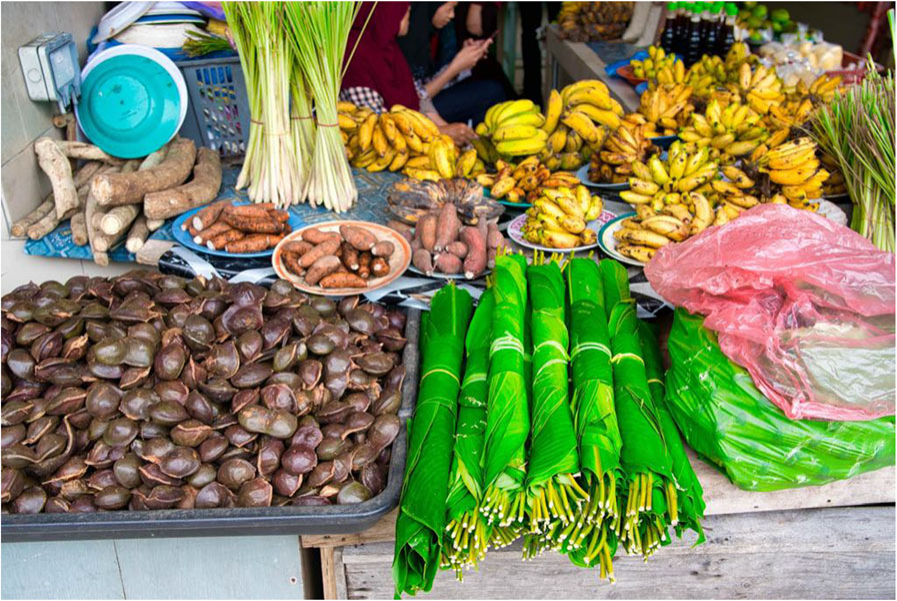
The diverse range of vegetables and fruits sold in Tamu Kianggeh is a major attraction. Credits: F. Merlin Franco

Our study shows that people visit Tamu Kianggeh for various reasons such as diversity of vegetables/fruits (*n* = 112; 70%), local fruits and vegetables (*n* = 79; 49.4%), socialising (*n* = 40; 25%), cheap prices (*n* = 91; 56.9%), ability to bargain (*n* = 38; 23.8%), freshness of the products (*n* = 78; 48.8%), convenience (*n* = 73; 45.6%), medicinal plants (*n* = 20; 12.5%), snacks (*n* = 75; 46.9%) and others. When asked to pick one reason among the list, respondents ranked availability of a wide range of fruits and vegetables as the highest, with 90 respondents (56.3%) picking it as the main reason for visiting the market. The onetime inventorying of vegetables and fruits sold at Tamu Kianggeh and the nearest supermarket yielded 138 taxa (Table 3). Bananas, plums, pear and grapes could be identified only to the genus level in both these markets. Accounting for the fact that there could be considerable variations within these taxa unidentified beyond the level of genus, it is possible that the actual number of taxa could be higher. Tamu Kianggeh had relatively higher diversity with 104 taxa, compared to the 85 taxa sold in the supermarket (Fig. 3). We recorded 30 taxa including two endemics natively distributed in Borneo, of which 26 were recorded from Kianggeh and 14 from the supermarket. The two endemics were sold in the market while the supermarket had only one that is in popular demand in Brunei (*Durio kutejensis*)*.* Perhaps this is an indication of supermarkets to compete with the markets in bringing popular local fruits to the people [47, 48]. At present, Tamu Kianggeh provides a wider range of fruits and vegetables and also a higher percentage of native ones. This agrees with the results of the interviews that the availability of a diverse range of vegetables/fruits, and local fruits and vegetables are major attractions of markets. However, it should also be borne in mind that the local people’s perception of local plants could differ from formal biogeographical understanding of it. Food plants could be cultivated thousands of kilometres away from their centres of origin, and any crop cultivated by the previous few generations could be considered ‘local’ by the people. Likewise, plants that are not native to the region could also get incorporated into the local cultures. Examples are: *Mangifera caesia* that is not native to Borneo is cultivated widely and considered a ‘local fruit’ in the region [49] and *Cosmos caudatus* that is native to tropical America, but popularly used in the local cultures of Malesia for its medicinal and culinary properties [50] and even depicted in the $10 bill of Brunei Darussalam. Although our study does not trace the habitats from where the vegetables and fruits are sourced, we do not rule out the possibility of fruits in the markets and supermarkets originating from home-gardens and forest gardens of the region [28, 51].

**Table 3:** Onetime inventory of taxa traded in Tamu Kianggeh and nearest supermarket

Sl. No.	Scientific name	Common name (English)	Common name (Others)	Native range	Tamu Kianggeh	Supermarket
1.	*Abelmoschus esculentus* (L.) Moench	Lady’s finger	Bendi	Exotic. Old World tropics	Yes	–
2.	*Actinidia deliciosa* (A.Chev.) C.F.Liang & A.R.Ferguson	Kiwi	–	Exotic. China	–	Yes
3.	*Allium ampeloprasum* L.	Leek	–	Exotic. Southern Europe to Western Asia	Yes	Yes
4.	*Allium ascalonicum* L.	Shallot (fruit, leaves)	Bawang merah; Daun bawang	Exotic. Central Asia	Yes	Yes
5.	*Allium cepa* L.	Onion (large, bombay, brown)	Bawang basar, Bawang Bombay	Not known in wild	Yes	Yes
6.	*Allium sativum* L.	Garlic	Bawang putih	Native. Asia	Yes	Yes
7.	*Allium tuberosum* Rottler ex Spreng.	Garlic chives	Kucai	Native. Southeast Asia	Yes	Yes
8.	*Aloe vera* (L.) Burm.f.	Aloe vera	Lidah buaya	Exotic. Mediterranean	–	Yes
9.	*Alpinia galangal* (L.) Willd.	Galangal	Lengkuas	Native. Southeast Asia	Yes	Yes
10.	*Alpinia purpurata* (Vieill.) K.Schum.	Red ginger	Halia bara	Exotic. Maluku to SW Pacific	Yes	*–*
11.	*Amaranthus blitum* L.	Spinach	Bayam itik, Bayam padi	Exotic. Peru to Brazil and N. Argentina	Yes	–
12.	*Amaranthus hybridus* L.	Green Spinach	Bayam hijau	Exotic. Tropical and subtropical America	–	Yes
13.	*Amaranthus tricolour* L.	Spinach	Bayam merah, Bayam hati	Exotic. Africa, Indo-China	Yes	–
14.	*Anacardium occidentale* L.	Cashew (shoots)	Pucuk jagus	Exotic Trinidad to S. tropical America	Yes	–
15.	*Ananas comosus* (L.) Merr.	Pineapple	Nenas	Exotic. Brazil	Yes	Yes
16.	*Annona muricata* L.	Soursop	Durian salat, Durian belanda	Exotic. Central America, West Indies	Yes	Yes
17.	*Annona reticulata* L.	Custard apple	Buah nona	Exotic. Caribbean, Central America	Yes	–
18.	*Apium graveolens* L.	Celery	Celery	Exotic. Europe	Yes	Yes
19.	*Archidendron jiringa* (Jack) I. C. Nielsen	–	Jering	Native. Bangladesh to Jawa	Yes	–
20.	*Arctium* spp.	Burdock	–	Exotic. Europe to Asia	–	Yes
21.	*Areca catechu* L.	Areca nut	Buah pinang	Exotic. Philippines	Yes	–
22.	*Artocarpus integer* (Thunb.) Merr.	–	Tibadak	Native. Sumatera to New Guinea	Yes	–
23.	*Artocarpus odoratissimus* Blanco	–	Tarap	Native. Borneo to Philippines	Yes	–
24.	*Asparagus officinalis* L.	Asparagus	–	Exotic. Europe and temperate Asia	–	Yes
25.	*Averrhoa bilimbi* L.	Sour starfruit	Belimbing buluh, Belimbing pucung	Exotic. Laos, Malaya, Maluku, Myanmar, Sulawesi	Yes	–
26.	*Averrhoa carambola* L.	Starfruit	Belimbing	Exotic. Jawa, Laos, Philippines, Sulawesi	Yes	Yes
27.	*Baccaurea macrocarpa* (Miq.) Müll.Arg.	–	Tampoi	Native. Peninsula Thailand to W. Malesia.	Yes	–
28.	*Baccaurea motleyana* (Müll.Arg.) Müll.Arg.	–	Rambai	Native. Peninsula Thailand to W. Malesia.	Yes	–
29.	*Bambusa xueana* Ohrnb.	Bamboo sprout	Rebung	Exotic. China	–	Yes
30.	*Basella alba* L.	Malabar spinach	Tandula, Gendola, Pacar Pindula, Wang Miu, Bayam Bangala; Bayam Taiwan	Native. Tropical Asia	Yes	Yes
31.	*Benincasa hispida* (Thunb.) Cogn.	Winter melon, wax gourd	Kundur, Gambas	Native. Central Malesia to SW. Pacific	Yes	Yes
32.	*Beta vulgaris* L.	Beetroot	Ubi bit, Daun ubi bit	Exotic. Europe	Yes	–
33.	*Brassica juncea* (L.) Czern.	Cabbage, Mustard	Sawi pahit, Kai chye	Exotic. Russia to central Asia	Yes	Yes
34.	*Brassica oleracea* L.	Chinese kale, Broccoli, Cabbage, Cauliflower	Kai lan, kubis	Exotic. Mediterranean region and southwestern Europe	Yes	Yes
35.	*Brassica rapa* L.	Celery cabbage	Sawi putih, Pak choi, Sawi bunga, Sawi manis, Sawi Taiwan, Choi sem, Chye sim, Pak chye, Yu mark	Exotic. Central and E. Mediterranean to W. Asia	Yes	Yes
36.	*Calamus peregrinus* Furtado	–	Asam jelayan	Exotic. S. Myanmar to Peninsula Malaysia.	Yes	–
37.	*Canarium odontophyllum* Miq.	–	Dabai	Native	Yes	–
38.	*Capsicum annuum* CV group *longum*	Chilli, Capsicum	Lada Bangala; Lada hidup	Exotic. Tropical North and South America	Yes	Yes
39.	*Capsicum annuum* L.	Chilli	Lada padi; Lada padi bara; Lada susu; Lada Thailand	Exotic. Tropical North and South America	Yes	Yes
40.	*Carica papaya* L.	Papaya	Betik	Exotic. S. Mexico to Venezuela	Yes	Yes
41.	*Centella asiatica* (L.) Urb.	Pennywort	Pegaga	Native. Caucasus, Tropical and Subtropical Old World to New Zealand and SW. Pacific	–	Yes
42.	*Citrullus lanatus* (Thunb.) Matsum. & Nakai	Watermelon	Sikui	Exotic. North Africa	Yes	Yes
43.	*Citrus assamensis* R.M.Dutta & Bhattacharya	Orange	Locsweet oren	Exotic. Assam to Bangladesh	Yes	Yes
44.	*Citrus aurantiifolia* (Christm.) Swingle	Lime, Key	Limau kapas	Possible hybrid	Yes	–
45.	*Citrus hystrix* DC.	Leech (fruit, leaves)	Limau purut	Native. China to Indo-China and New Guinea, Wallis Island	Yes	Yes
46.	*Citrus limon* (L.) Osbeck	Lemon	–	Native. Asia	Yes	Yes
47.	*Citrus maxima* (Burm.) Merr.	Pomelo, shaddock	Limau basar, Limau Bali	Exotic. Polynesia	–	Yes
48.	*Citrus reticulata* Blanco	Orange	Limau mandarin, Limau madu; Limau manis	Exotic. Other parts of Southeast Asia	Yes	Yes
49.	*Cocos nucifera* L.	Coconut (fruit, shoots)	Kelapa	Exotic. Central Malesia to SW. Pacific	Yes	–
50.	*Colocasia esculenta* (L.) Schott	Yam (root, shoot)	Keladi, Ubi belayar	Exotic. India to S. China and Sumatera	Yes	–
51.	*Cosmos caudatus* Kunth	–	Ulam raja, Rancah—rancah	Exotic. Mexico to S. Tropical America	Yes	–
52.	*Cucumis melo* L.	Hami melon, Musk melon, Honeydew, Beloro (fruit, shoot), Fragrant cucumber	Betat, Timun batat, Timun suri	Exotic. Ethiopia to S. Africa, Arabian Peninsula to India, N. and Central Australia	Yes	Yes
53.	*Cucumis sativus* L.	Cucumber (fruit, shoot)	Timun	Exotic. Himalaya to N. Thailand	Yes	Yes
54.	*Cucurbita ficifolia* Bouché	Sharkfin melon		Exotic. Peru to Bolivia	Yes	Yes
55.	*Cucurbita moschata* Duchesne	Pumpkin (fruit, shoot)	Labu	Exotic. Mexico to Guatemala	Yes	Yes
56.	*Cucurbita pepo* L.	Zucchini	–	Exotic. North America	–	Yes
57.	*Curcuma longa* L.	Turmeric (root, leaves)	Kunyit	Exotic. India, Malaysia	Yes	Yes
58.	*Cymbopogon citratus* (DC.) Stapf	Lemongrass	Serai	Exotic. Southern India, Sri Lanka	Yes	–
59.	*Daucus carota* L.	Carrot, orange/purple	Lobak	Exotic. Afghanistan	Yes	–
60.	*Dialium indum* L.	–	Keranji	Native. Thailand to W. Malesia	Yes	Yes
61.	*Dimocarpus longan* Lour.	Longan	–	Native.		Yes
62.	*Dimocarpus longan* subsp. *malesianus* Leenh.	–	Mata kucing	Native.	Yes	–
63.	*Dioscorea polystachya* Turcz.	Chinese yam (Huai Sun)	–	Exotic. Central and S. China to Kuril Islands and Taiwan	–	Yes
64.	*Diospyros kaki* L.f.	Persimmon	Pisang kaki, Kesamak	Exotic. Assam to Central and S. China and Taiwan	Yes	Yes
65.	*Durio kutejensis* (Hassk.) Becc.	Durian	Durian pulu	Endemic to Borneo	Yes	Yes
66.	*Durio zibethinus* L.	Durian	Durian monthong, Durian kawin	Native. Sumatera to Borneo	Yes	–
67.	*Eleiodoxa conferata* (Griff.) Burret	–	Asam kelumbi	Native. Thailand, Malaysia, Borneo and Sumatra	Yes	–
68.	*Eleocharis dulcis* (Burm.f.) Trin. ex Hensch.	Water chestnut	Kacang berangan	Exotic. Tropical and Subtropical Old World	–	Yes
69.	*Etlingera elatior* (Jack) R. M. Sm.	Torch ginger; Ginger flower	Bunga kantan	Native. Peninsula Thailand to W. Malesia	Yes	–
70.	*Garcinia mangostana* L.	Mangosteen	Manggis	Exotic. Peninsula Malaysia	Yes	–
71.	*Garcinia parvifolia* (Miq.) Miq.	Brunei cherry	Asam aur-aur	Native. W. and Central Malesia	Yes	–
72.	*Gnetum gnemon* L.	–	Bagu	Native. SE. Tibet to W. Pacific	Yes	–
73.	*Hylocereus costaricensis* (F.A.C. Weber) Britton & Rose	Dragon fruit, red	Buah naga	Exotic. South America	Yes	Yes
74.	*Hylocereus undatus* (Haw.) Britton & Rose	Dragon fruit, white	Buah naga (putih)	Exotic. Mexico to Columbia	–	Yes
75.	*Ipomoea aquatica* Forssk	–	Kangkong	Native. Tropical and Subtropical Old World	Yes	Yes
76.	*Ipomoea batatas* (L.) Lam.	Potato, sweet purple/orange/white (shoot)	Ubi manis, Jalar keladi	Exotic. Mexico	Yes	Yes
77.	*Kaempferia galanga* L.	Aromatic ginger	Cakur, Cekur	Exotic. China and Indo-China	Yes	–
78.	*Lactuca sativa* L.	Lettuce, Coral; Iceberg; Sword; Curly; Thailand Lettuce, Butterhead Cos lettuce, Romaine lettuce	Yu Ma, Selada keriting - Curly Lettuce	Exotic. West Asia	Yes	Yes
79.	*Lagenaria siceraria* (Molina) Standl.	Calabash gourd	Labu air, Labu putih	Exotic. W. Tropical Africa to Ethiopia and Tanzania	Yes	–
80.	*Lansium parasiticum* (Osbeck) K.C.Sahni & Bennet	–	Duku; langsat	Native. Taiwan (Lan Yü), Malesia to N. and NE. Queensland	Yes	–
81.	*Laurus nobilis* L.	Bay leaf	Daun salam, Daun kapau	Exotic. Northern Africa, Western Asia, Southern Europe	Yes	–
82.	*Luffa acutangula* (L.) Roxb.	Angled gourd	Petola; Petola gantang	Exotic. Indian sub-continent	Yes	Yes
83.	*Malus pumila* Mill	Apple (Red, Green)	Epal	Exotic. Central Asia to Afghanistan	Yes	Yes
84.	*Mangifera caesia* Jack	–	Belunu, Binjai	Exotic. Sumatera, Philippines and Lesser Sunda Island	Yes	–
85.	*Mangifera indica* L.	Mango	Mangga, Mangga mahathir, Dok mai, Mangga Thai, Mempalam, Manga apple	Exotic. Assam to China (S. Yunnan)	Yes	Yes
86.	*Mangifera pajang* Kosterm.	–	Mambangan	Endemic to Borneo	Yes	–
87.	*Manihot esculenta* Crantz	Cassava	Ubi kayu, Ubi kayu mentega, Ubi keriting	Exotic. W. South America to Brazil	Yes	Yes
88.	*Manilkara zapota* (L.) P.Royen	Sapota, Chikoo, Ciku	Ciku	Exotic. Mexico to Colombia	Yes	–
89.	*Maranta arundinacea* L.	Arrowroot	–	Native. Taiwan, Indo-China to W. Pacific	–	Yes
90.	*Mentha spicata* L.	Mint	Daun pudina	Exotic. Europe to China	–	Yes
91.	*Momordica charantia* L.	Bitter gourd	Peria buaya	Exotic. Tropical and Subtropical Old World	Yes	–
92.	*Momordica dioica* Roxb. ex Willd.	Spiny gourd, Teasel	Kakrol	Exotic. Other parts of Tropical Asia	Yes	–
93.	*Morinda citrifolia* L.	Morinda, Noni	Mengkudu	Exotic. India	Yes	–
94.	*Musa* spp.	Banana (Fruit, Blossom, Stem)	Pisang	–	Yes	Yes
95.	*Nasturtium officinale* R.Br.	Watercress	–	Exotic. Eurasia, Macaronesia, Tropical Africa	–	Yes
96.	*Nelumbo nucifera* Gaertn.	Lotus (Root, Seed)	–	Exotic. India, Bangladesh, Laos, China, Vietnam, etc.	–	Yes
97.	*Nephelium ramboutan-ake* (Labill.) Leenh.	–	Pulasan	Native. Native to Asia-Tropical	Yes	–
98.	*Ocimum basilicum* L.	Basil	Kemangi	Native. Tropical Asia	Yes	–
99.	*Pachyrhizus erosus* (L.) Urh.	–	Sengkuang	Central America, South America	Yes	–
100.	*Pandanus amaryllifolius* Roxb.	–	Pandan	Exotic. Maluku	Yes	–
101.	*Pangium edule* Reinw.	–	Kepayang	Exotic. Asia-Tropical, Vanuatu and Santa Cruz Island	Yes	–
102.	*Passiflora edulis* Sims	Passion fruit	–	Exotic. Brazil to NE. Argentina	Yes	Yes
103.	*Persea americana* Mill.	Avocado	–	Exotic. Central America	Yes	Yes
104.	*Petroselinum crispum* (Mill.) Fuss	Parsley (Chinese, English)	Daun Sup	Exotic. Balkan peninsula	Yes	Yes
105.	*Phaseolus vulgaris* L.	French bean, Red streaked bean	Kacang Merah	Exotic. Central and South America	Yes	–
106.	*Phoenix dactylifera* L.	Dates	Kurma	Exotic. Arabian Peninsula to S. Pakistan	–	Yes
107.	*Phyllanthus acidus* (L.) Skeels	–	Ceramai	Exotic. Brazil	Yes	–
108.	*Piper betle* L.	Betel (leaves)	Sirih	Exotic. Tropical Asia	Yes	–
109.	*Pisum sativum* L.	Snow peas, sweet peas	–	Southern Europe	Yes	–
110.	*Pometia pinnata* J.R.Forst. & G.Forst.	Matoa	Longan Brazil	Native. Sri Lanka to China (Yunnan) and S. Pacific	–	Yes
111.	*Pouteria campechiana* (Kunth) Baehni	Egg fruit	Buah keju	Exotic. Mexico to Central America	–	Yes
112.	*Prunus avium* (L.) L.	Cherry (Red, White)	Ceri	Exotic. Europe to Caucasus, Mediterranean to Iran	–	Yes
113.	*Prunus persica* (L.) Batsch	Peach	–	Exotic. N. Central China	–	Yes
114.	*Prunus* subg. *Prunus*	Plum	–	–	–	Yes
115.	*Psidium guajava* L.	Guava	Biabas	Exotic. Brazil	–	Yes
116.	*Psophocarpus tetragonolobus* (L.) DC.	Winges beans, square beans	Kacang sirik, Kacang belimbing	Exotic. New Guinea	–	Yes
117.	*Punica granatum* L.	Pomegranate	Delima	Exotic. NE. Turkey to Afghanistan	–	Yes
118.	*Pyrus* spp.	Pear	–	–	–	Yes
119.	*Raphanus raphanistrum* L.	Radish	Lobak	Exotic. Europe, Africa and Asia-Temperate	Yes	–
120.	*Raphanus raphanistrum subsp. sativus* (L.) Domin	Radish	Lobak	Exotic. Greece, Gulf States, Italy, Oman, Sicilia, Tadzhikistan, Balkan peninsula	–	Yes
121.	*Sagittaria latifolia* Willd.	Arrow head; Arrow shoot	–	Exotic. Eastern and central North America	–	Yes
122.	*Sauropus androgynus* (L.) Merr.	Cangkok manis	–	Native. Tropical and Subtropical Asia	–	Yes
123.	*Sechium edule* (Jacq.) Sw.	Chayote gourd	Labu Siam; Timun duri	Exotic. Mexico to Belize	Yes	Yes
124.	*Solanum aethiopicum* L.	Dayak eggplant	Terung Iban; Terung asam; Sesaie Iban	Exotic. NE. Tropical Africa	Yes	–
125.	*Solanum lycopersicum* L.	Tomato	Tomat	Exotic. Peru	Yes	Yes
126.	*Solanum melongena* L.	Eggplant	Terung, Terong Korea	Exotic. Laos, Myanmar, Vietnam	Yes	Yes
127.	*Solanum tuberosum* L.	Potato	Ubi; Ubi Thailand	Exotic. South America	Yes	Yes
128.	*Solanum undatum* Lam.	Thailand eggplant	Terung Thailand	Exotic. W. Indian Ocean, Tropical and Subtropical Asia	–	Yes
129.	*Spondias dulcis* Parkinson	June plum	Kedondong	Native. Malesia to Santa Cruz Islands. Only known in cultivation	Yes	–
130.	*Syzygium malaccense* (L.) Merr. & L.M. Perry	Rose apple	Jambu merah	Exotic. Indo-China to Vanuatu	–	Yes
131.	*Tamarindus indica* L.	Tamarind	Asam Jawa	Exotic. Madagascar	–	Yes
132.	*Telosma cordata* (Burm. f.) Merr.	–	Bunga tongkeng	Exotic. Pakistan to China	Yes	–
133.	*Vigna radiata* (L.) R. Wilczek	Bean sprout	Taugeh	Exotic. Indian sub-continent to Myanmar	Yes	Yes
134.	*Vigna unguiculata* (L.) Walp.	Snake bean	Kacang ular, Kacang keriting	Exotic. Africa	Yes	–
135.	*Vigna unguiculata subsp. sesquipedalis* (L.) Verdc.	Long beans (flower, fruits, leaves)	Kacang panjang	Exotic. Africa	Yes	Yes
136.	*Vitis* spp.	Crimson grapes	Anggur	–	–	Yes
137.	*Zea mays* L.	Corn	Jagung	Exotic. Mexico, Guatemala	Yes	Yes
138.	*Zingiber officinale* Roscoe	Ginger	Halia	Exotic. India and China	Yes	Yes

People visiting local markets are indeed known to show preferences for fruits and vegetables [52]. A study with a New Jersey farmers’ market shows that close to 80% of consumers reported an increase in consumption of fresh fruits, and 78% increased consumption of vegetables over a period of 5 years [53]. The study of Baker et al. on the markets of north-western Vermont showed that people visited the markets primarily for the local and ‘fresh’ food, which in our study is ranked as the second most important reason [23]. These show that markets play an important role in bringing biodiversity closer to people, while also contributing to enhanced food diversity. Although some respondents listed medicinal plants (12.5%) as one of the reasons to visit the market, none of them chose it as a primary reason, indicating that purchase of medicinal plants is not the primary purpose of visit. Medicinal plants sold in markets are known to have high informant consensus, due to the cultural selection and demand for plants with high efficacy [54]. Studies have also shown that medicinal plants sold in markets could be locale and market specific [55]. The practice of using traditional medicine is highly prevalent in Brunei [56]; hence, the contribution of the market towards local healthcare in Brunei cannot be under-estimated. Medicinal plants were not included in the onetime plant survey we had conducted. We identify this as major gap in the current study and recommend that future studies should look into the diversity of medicinal plants traded in the markets of Brunei Darussalam.

The results presented in this section show that biodiversity represented by the diverse range of fruits and vegetables is the major reason for the patronage showed by people towards Tamu Kianggeh. It is also noteworthy that availability of local fruits and vegetables (*n* = 79; 49.4%) is ranked second. From these findings, it is inferred that Tamu Kianggeh brings people closer to the local biodiversity. One of the greatest tragedy of our times is ‘plant blindness’, our inability to recognise the plants in our environment [57]. The major reason for such blindness, especially in urban ecosystems is the inability to stay in continuous contact with biodiversity [58]. From a ‘Biocultural Ethics’ perspective, it is important to surpass such hurdles by revitalising the links between people and biodiversity [58, 59]. Balding and Williams are of the opinion that being in a ‘plant culture’ enhances an individual’s ability to ‘detect, recall, and value plants’ [60]. Likewise, creating interest in useful plants has been proposed as the first step for developing interest in the plant kingdom [59]. Open-air markets provide people opportunities to be immersed in a plant culture where socialising happens in the company of useful plants. In a recent paper, Krishnan et al. [5] reiterate the need for collaborations between botanic gardens, academic institutions, non-governmental bodies and research institutes to combat plant blindness related to food plants. Our results show that such efforts should also include local markets as they are cultural spaces where people come into close contact with biodiversity represented by fruits, vegetables and medicinal plants.

### Markets and cultural diversity

In the preceding section, we showed that visitors find biodiversity as the major attraction at Tamu Kianggeh. Are markets mere trading centres where people procure products of biodiversity? Anthropologists consider markets as an integral part of complex societies [61]. Beyond being essential trading centres, they are also natural units of social interaction—an important biocultural feature that is often overlooked [17]. In our sample, there were 54.4% (*n* = 87) of respondents who identified themselves as a member of an ethnic group indigenous to Borneo, while 45.6% (*n* = 73) identified themselves as non-indigenous. Majority of the respondents who identified themselves as indigenous also identified themselves as a member of the Malay community (*n* = 58, 66.7%), followed by Kedayan (*n* = 17, 19.5%), Iban (*n* = 5, 5.7%), Dusun (*n* = 3, 3.5%), Tutong (*n* = 3, 3.5%) and Sarawak Kuching (*n* = 1, 1.1%). There is no formal ethnic community such as ‘Sarawak Kuching’, and the lone respondent refused to name his community and just mentioned that he is an indigenous member from Kuching in Sarawak. In addition, there were also a significant percentage of foreigners of unknown ethnicities visiting the market (*n* = 50, 31.2%). This shows that Tamu Kianggeh is also a space where people of diverse ethnic groups meet and socialise, an aspect that is often overlooked [62]. The confluence of multiple ethnicities could be considered indicative of the representation of multiple languages [63]. Hidayati et al.’s research with the Vaie people of Malaysian Borneo illustrates how local markets are important centres of transmission for local languages and traditional knowledge [63]. Their research also shows that markets are spaces where new lexemes are coined, a phenomenon which affects language diversity and maintenance.

Our study shows that none of the visitors arrived at the market with the primary intention of socialising, although a significant percentage (25%, *n* = 40) of them see ability to socialise as one of the reasons to visit the market. However, 40.6% (*n* = 65) of the respondents purchased from a regular vendor indicating trust-based relationships developed through regular visits. The study of Watson and Studdert [64] and Alexander [20] show that it is the vendors with a long history in the markets who play an important role in attracting customers. In the current study, majority of those who tend to purchase from regular vendors are either regular market visitors (*n* = 46, 70.8%) or locals (*n* = 46, 70.8%).

There was a significant difference between regular and non-regular respondents (*p* = 0.037) with regard to their intention to socialise. Of the respondents who reported an intention to socialise while visiting the market, 77.5% (*n* = 33) were regular visitors. There were no significant differences in the intention to socialise between foreigners and locals (*p* = 0.846). Although majority of the respondents (*n* = 97; 60.6%) do not visit the market with friends, the fact that the remaining 39.4% of the respondents did visit the market with friends either regularly or occasionally indicates that markets do provide opportunities to strengthen socialising between already existing friendships. Likewise, a large number of respondents (*n* = 97; 60.6%) reported that they had made friends at the market, further indicating the role of open-air markets as platforms for finding new friends. Results also show that majority (*n* = 84; 86.6%) of such friendships were reported to be formed with vendors, while friendships with other visitors also formed a significant percentage (*n* = 32; 33%). Among those who formed friendships with visitors, the proportion of non-indigenous respondents who responded affirmatively (*n* = 15; 41.7%) was comparatively higher than those who reported themselves as indigenous (*n* = 17; 27.9%). In some cultures such as the tribal societies of Odisha in India, markets are also spaces for courtship and socialising with the opposite gender [65]. However, our study did not deal with such aspects of socialising due to cultural constraints.

Majority of the respondents (*n* = 89; 55.6%) reported that their neighbours do not ask them for favours of purchasing items from the market. People who reported ‘sometimes’ also formed a significant percentage (*n* = 48; 30.0%), while there was a relatively small percentage of respondents whose neighbours do approach them for such favours (*n* = 23, 14.4%). This indicates that markets facilitate socialising happening beyond the actual market place. A significant number of respondents (*n* = 109; 68.1%) reported that they would not be visiting the market if they do not have to purchase anything, while 31.9% of the respondents (*n* = 51) were of the view that they would be visiting the market to roam around, even if they do not have to purchase anything. Such leisure visitors (*n* = 25; 49%) are more likely to visit the market with their pre-existing friends than visitors with a purpose (*n* = 38; 34.9%). Leisure visitors also have the tendency to be approached by neighbours for a favour from the market (*n* = 25; 49%) than the visitors with a purpose (*n* = 46; 42.2%).

The study of Watson and Studdert [64] shows that foreigners also use markets as important spaces for socialising. In Kianggeh, the likelihood of a foreigner visiting the market for leisure purposes appears to be increasing with the duration of residency as 35.7% (*n* = 5) of foreigners who have resided in the city for 1–5 years were likely to visit the market for leisure purposes, while the proportion for those who have resided for more than 5 years is significantly higher at 57.1% (*n* = 8). However, the proportion of foreigners who visited the market for purchase purposes also increased with the year of residency from 11.1% (*n* = 4) in the 1–5 year group to 83.3% (*n* = 30) in the above 5 years group. This only shows that people are more likely to visit the market with increasing years of residency. There were only two respondents who identified themselves as tourists; among them, one had visited the market for leisure purposes while the other was there to purchase. Thirty eight of the 50 foreigners who agreed to participate in our study (76%) reported to visit the market every week. Of them, 28 (73.6%) also regularly patronise local markets in their native country. This indicates a continuous cultural affinity towards open-air markets despite migration.

These findings presented in this section have implications for the field of Biocultural Diversity that espouses the ‘inextricable’ link between biodiversity and cultural diversity [1, 2, 66]. The results show that although the market’s primary purpose is trading biodiversity, it also serves as a platform for convergence of people of multiple ethnicities. In addition, the market is an important recreational and cultural space for people to socialise. Another noteworthy finding is the increasing interest of foreigners in visiting the local market along with the increase in years of residency. This could be considered indicative of the ability of local markets in attracting foreigners towards local plant culture.

### Open-air markets are irreplaceable

One of the important features of urban centres is the presence of supermarkets. Supermarkets differ from open-air markets in the ownership, formal versus informal nature and cultural contributions to the society. Yet it is common to see open-air markets throughout the urban centres of Southeast Asia which indicates the patronage they receive despite urbanisation. Majority of our respondents (*n* = 153; 95.6%) were of the opinion that every city should have an open-air market, and there were no significant differences between indigenous and non-indigenous respondents (*p* = 0.081). Almost equal number of respondents agreed in favour or against the statement that there are groceries that could be exclusively found in the open-air markets when compared to supermarkets. However, the percentage of those who agreed with the statement was significantly higher (*p* = 0.007) for the indigenous respondents (*n* = 53; 60.9%) when compared to the non-indigenous groups (*n* = 28; 38.4%). Majority of the non-indigenous respondents (*n* = 45; 61.6%) disagreed with the statement. This indicates that the nature of dependence on the market is different for the indigenous and non-indigenous people. Of the respondents who responded affirmatively, majority (*n* = 54; 81.1%) had listed ‘local goods’ as the item traded exclusively in the market, irrespective of their indigenous/non-indigenous nature. On the other hand, imported (*n* = 33; 47.1%) and processed (*n* = 33; 47.1%) goods were ranked high in the list of exclusive goods available in the supermarkets, while local products were little (*n* = 2; 2.9%). Minten and Reardon (2008) explain this phenomenon of supermarkets specialising in processed food using the three wave concept. Supermarkets in their early stages of market penetration tend to offer competitively priced processed and packages foods, while fresh produces including vegetables are sold at rates expensive than ‘traditional retailers’ including markets. However, they gradually encroach into the consumer base of the traditional retailers and markets by offering competitively priced fresh fruits and vegetables. In the case of Kianggeh, we could consider the availability of the endemic durian in the supermarket as an indicator of the intentions of the supermarket to compete with the market in offering local produces. However, for indigenous and local people, open-air markets shall continue to be irreplaceable. This is understandable from their univocal response that all cities should have open-air markets.

### Limitations of the study

The study involves visitors of Tamu Kianggeh and thus may not be reflective of other markets in Brunei. Likewise, BSB is relatively smaller to other major cities of Asia such as Kuala Lumpur or Bangkok and hence cannot be considered generalizable to other countries in Asia. The inventory of fruits and vegetables traded in the market has been undertaken only once. Some of these produces could be seasonal and, ideally, surveys of traded fruits and vegetables should be undertaken throughout the year on a monthly basis. The sample consisted of visitors alone, and does not provide an understanding of the reasons for the popularity of the market from the vendors’ point of view. We suggest the readers consider the findings of the study in the context of the above limitations.

## Conclusion

Our study shows that open-air markets are meeting grounds of ethnic and biological diversities, a property that makes them important nodes of biocultural diversity at the local level. Tamu Kianggeh is a meeting point for multiple ethnicities such as the Malay, Kedayan, Iban, Dusun, Tutong and Chinese. Besides, there is also a significant percentage of foreigners who visit Kianggeh, majority of whom also reported to be a frequent visitor to the market in their home country. This indicates a continuum in the cultural preference towards local markets, despite migrating to a new country. Our study also reports the increasing interest of foreigners in visiting the local market along with the increase in years of residency. This indicates of the ability of local markets in attracting foreigners towards local biodiversity and plant culture. People patronise Tamu Kianggeh for the wide range of vegetables and fruits sold there, followed by the prospects of finding local fruits and vegetables. True to the results of the survey, our study finds that the market sells higher number of taxa than the nearest supermarket. Availability of medicinal plants is also one of the reasons for people visiting the market, indicating its relevance in local knowledge and healing. The market also sells a higher percentage of native taxa including two endemics. The findings show that open-air markets such as Tamu Kianggeh bring people closer to each other as well as to the local biodiversity. Krishan et al. [5] suggest ‘exhibits, demonstration farms, experiential education, community outreach and collaborative biocultural conservation’ as measures to enhance people’s ties with food plants. We propose that such measures should also include open-air markets.

## Data Availability

The dataset supporting the conclusions of this article is included within the article.

## Abbreviations

BCD: Biocultural diversity
Sp(p): Species
